# Genotoxicity of styrene oligomers extracted from polystyrene intended for use in contact with food

**DOI:** 10.1016/j.toxrep.2014.11.007

**Published:** 2014-11-15

**Authors:** Makoto Nakai, Mifumi Tsubokura, Masaru Suzuki, Saori Fujishima, Yoichi Watanabe, Yu Hasegawa, Kazuhiro Oyama, Shozo Ogura

**Affiliations:** a Chemicals Evaluation and Research Institute, Japan, CERI Hita, Hita-shi, Oita, Japan; b Chemicals Evaluation and Research Institute, Japan, CERI Kurume, Kurume-shi, Fukuoka, Japan

**Keywords:** EFSA, European Food Safety Authority, GPPS, general purpose polystyrene, FDA, United States Food and Drug Administration, OECD, Organisation for Economic Co-operation and Development, SD, styrene dimer, ST, styrene trimer, Food packaging, *In vitro* genotoxicity test, Styrene oligomers

## Abstract

Here, we conducted *in vitro* genotoxicity tests to evaluate the genotoxicity of styrene oligomers extracted from polystyrene intended for use in contact with food. Styrene oligomers were extracted with acetone and the extract was subjected to the Ames test (OECD test guideline No. 471) and the *in vitro* chromosomal aberration test (OECD test guideline No. 473) under good laboratory practice conditions. The concentrations of styrene dimers and trimers in the concentrated extract were 540 and 13,431 ppm, respectively. Extraction with acetone provided markedly higher concentrations of styrene oligomers compared with extraction with 50% ethanol aqueous solution, which is the food simulant currently recommended for use in safety assessments of polystyrene by both the United States Food and Drug Administration and the European Food Safety Authority. And these high concentrations of styrene dimers and trimers were utilized for the evaluation of genotoxicity *in vitro*. Ames tests using five bacterial tester strains were negative both in the presence or absence of metabolic activation. The *in vitro* chromosomal aberration test using Chinese hamster lung cells (CHL/IU) was also negative. Together, these results suggest that the risk of the genotoxicity of styrene oligomers that migrate from polystyrene food packaging into food is very low.

## Highlights

• We evaluated the genotoxicity of styrene oligomers extracted from polystyrene intended for use in contact with food.
• Compared with 50% ethanol, acetone extracted a far greater amount of styrene dimers and trimers from polystyrene.
• Ames tests and an *in vitro* chromosomal aberration test were negative.
• The risk of the genotoxicity of styrene oligomers migrated from polystyrene food packaging into food is likely very low.

## 1 Introduction

Polystyrene was first used industrially in Germany in the mid-1930s, and since then, because of its useful characteristics such as high processability, shape reproducibility, and superior foaming ability, it has been widely used in the production and packaging of commodities such as electronic devices and food. It is estimated that in 2010 approximately 10.8 million tons of polystyrene was used worldwide [1]. In the developed world, 50–60% of the production volume of polystyrene is used for food packaging [1], [2]. Furthermore, polystyrene represents approximately 14% and 10% of plastic food-packaging materials used in the United States and Japan, respectively [3], [4]. Polystyrene is therefore an important material for the packaging of food.

Polystyrene products have been shown to contain styrene oligomers, which were generated as byproducts in the process of polymerization [5]. Concerns related to the human health effects of styrene dimers (SDs) and styrene trimers (STs) have been raised by some investigators [6]. Kawamura reported that several kinds of SDs and STs can migrate from polystyrene products [7], [8]. When using 50% of ethanol aqueous solution, 30–70 ppb of styrene trimers were extracted and no styrene dimers were detected [7]. Under intact usage, although no styrene dimers were detected, the amount of the migrated styrene trimers were estimated to be up to 33.8 μg for one meal [8]. The endocrine-disrupting potencies of SDs and STs were actively investigated in the late 1990s to early 2000s. Although there were some results indicating positive responses in *in vitro* tests [9], the results based on many reliable studies including *in vivo* studies suggested that SDs and STs do not have endocrine disrupting potential [10], [11], [12], [13]. For example, it was shown that SDs and STs extracted from polystyrene with acetone cause no reproductive toxicity in rats, either to dams or offspring, at concentrations of up to 1.0 mg/kg/day, which is a concentration 1000 times greater than the daily intake in humans [13].

Prior to new polymers being authorized for use in the United States, the safety of extracted compounds, including oligomers, must be assessed [3]. In the European Union, the regulations on plastic materials for food packaging were revised in 2011 [14], and when a new polymer is produced, the safety of unintentionally generated byproducts must be assessed. Thus, the safety of oligomers present in plastic food packaging is of great concern. Genotoxicity testing by means of the Ames test and the *in vitro* chromosomal aberration test are toxicological endpoints that are required by both the US Food and Drug Administration and the European Food Safety Authority [15].

Despite the importance of the genotoxic effects of styrene oligomers on human health, little information is currently available. However, Grifoll et al. [16] did report a negative Ames test for the genotoxicity of styrene oligomers in *Salmonella typhimurium* strain TA98 under conditions of metabolic activation.

Here, we evaluated the genotoxicity of styrene oligomers extracted from polystyrene with acetone by means of the Ames test and the *in vitro* chromosomal aberration test.

## 2 Materials and methods

### 2.1 Materials

General purpose polystyrene (GPPS) pellets were supplied by Japan Styrene Industry Association (Tokyo, Japan). The molecular characteristics of GPPS pellets used in this study were as follows: The number-average and weight-average molecular weights were 72,000 and 222,000, respectively, and the concentration of SDs and STs in the GPPS pellets were 0.16% (w/w) and 1.02% (w/w), respectively. *S. typhimurium* strains TA100, TA1535, TA98, and TA1537, and *Escherichia coli* strain WP2*uvrA* were purchased from National Institute of Technology and Evaluation (Tokyo, Japan). Chinese hamster lung fibroblasts (CHL/IU) were purchased from Health Science Research Resources Bank, Japan Health Sciences Foundation (Tokyo, Japan). S9 prepared from the livers of seven-week-old male Sprague-Dawley rats administered phenobarbital and 5,6-benzoflavone was purchased from Oriental Yeast Co. (Tokyo, Japan). All reagents were of the best grade available.

### 2.2 Methods

#### 2.2.1 Preparation of test samples

The test solution used in the genotoxicity tests was produced as follows: GPPS pellets (120 g) were added to 600 mL of acetone and the mixture was stirred with a Teflon-coated stir bar for 1 h at 40 °C. The solution was then mixed with 2.4 L of methanol, stirred for 1 h at room temperature, and then filtered through No. 2 filter paper (Toyo Roshi, Tokyo, Japan). The filtrate was evaporated (bath temperature, approx. 40 °C) and the residue was dissolved in acetone to prepare 20 mL of acetone solution. This concentrated solution was then used as the test solution in the genotoxicity tests and was considered to have a purity of 100%.

The concentrations of SDs and STs in the test solution were determined by means of gas chromatography–mass spectrometry. The analytical conditions are shown in Table 1.

**Table 1:** Gas chromatography–mass spectrometry conditions for the determination of the styrene dimers and trimers present in the test solution.

GC/MS System	Agilent 6890N/5973N gas chromatograph (Agilent Technologies, CA, USA)
Column	DB-WAX (0.25 mm × 30 m; film thickness, 0.25 μm; Agilent Technologies)
Column temperature	100–250 °C at 20 °C/min, hold at 100 °C for 1 min, and 300 °C for 5 min
Injection temperature	250 °C
Interface temperature	250 °C
Ion source temperature	230 °C
Ionization voltage	70 eV
Ionization method	Electronic ionization
Injection mode	Splitless
Injection volume	2 μL
Carrier gas	Helium

The molecular weight distribution of the test sample was determined by means of gel permeation chromatography. The analytical conditions are shown in Table 2. One milliliter of test sample was dried under a nitrogen gas purge and the residue was then dissolved in tetrahydrofuran to make 10 mL of tetrahydrofuran solution. The tetrahydrofuran solution was kept at 25 °C for approximately 24 h before use.

**Table 2:** High-performance liquid chromatography conditions for the determination of the molecular weight distribution of the test solution.

Instruments	
GPC system	GPC-101 (Showa Denko K.K., Tokyo, Japan)
Detector	Refractive Index Detector (Showa Denko K.K.)
Data treatment system	Shodex 480 II (Showa Denko K.K.)
Column	Shodex GPC KF-806 L × 2 (8.0 mm × 300 mm, Showa Denko K.K.)
	Shodex GPC KF-800D × 1 (8.0 mm × 100 mm, Showa Denko K.K.)
Column temperature	40 °C
Eluent	Tetrahydrofuran
Flow rate	0.8 mL/min
Injection volume	100 μL

GPC, gel permeation chromatography.

#### 2.2.2 Ames test

The Ames test was conducted according to the Organisation for Economic Co-operation and Development (OECD) Guideline for the Testing of Chemicals, No. 471, Bacterial Reverse Mutation Test [17], as follows.

##### 2.2.2.1 Chemical treatment and colony counting

A pre-incubation method in the presence or absence of S9 mix was used [18]. Triplicate plates were used for each dose. *S. typhimurium* strains TA100, TA1535, TA98, and TA1537 and *E. coli* strain WP2*uvrA* were used as the bacterial tester strains. The test solution was diluted with acetone to prepare the test doses. The maximum concentration of the test doses was 10% (w/v). The test sample formulation was mixed with the bacterial culture in the presence or absence of S9 mix and pre-incubated at 37 °C for 20 min. Soft agar was added to the mixture, which was then poured onto a minimal glucose agar plate (Tesmedia AN; Oriental Yeast Co., Tokyo, Japan). Triplicate plates were used for each dose. The final concentration of S9 in the top agar layer was 2%. After incubation at 37 °C for 48 h, the number of revertant colonies was counted by using a colony counter system (CA-11D; System Sciences, Tokyo, Japan). Precipitation of the test sample and inhibition of bacterial growth were also checked macroscopically. To confirm the reproducibility of the test results, two independent tests were conducted.

##### 2.2.2.2 Evaluation of results

The Ames test was considered positive when the number of revertant colonies was increased to two or more times that of the negative control and when the response was dose-related or reproducible, or both. All other cases were considered negative. No statistical methods were used.

#### 2.2.3 *In vitro* chromosomal aberration test

The *in vitro* chromosomal aberration test was conducted according to OECD Guideline for the Testing of Chemicals, No. 473, *In Vitro* Mammalian Chromosome Aberration Test [19], as follows.

##### 2.2.3.1 Chemical treatment, slide preparation, and assessment

The procedure reported by Ishidate and Odashima [20] was followed. CHL/IU cells were pre-cultured in 10% (v/v) heat-inactivated newborn calf serum/minimum essential medium in CO_2_ incubator (MCO-18AIC, SANYO Electric, Osaka, Japan), which was set at 37 °C and an atmosphere of 5% CO_2_ under a humid condition. Duplicate dishes were used for each dose. The test solution was diluted with acetone to prepare the test doses. The maximum concentration of the test dose was 50% (w/v). Cells were treated with medium containing 1% (v/v) of the test sample formulation. For short-term treatment, cells were treated for 6 h at 37 °C in the presence or absence of 5% S9, after which, the medium was discarded, and the cells were rinsed with PBS(−) and cultured for another 18 h at 37 °C in fresh medium. For continuous treatment, cells were treated for 24 h in the absence of metabolic activation. At the start and end of the treatment period, and at the end of culturing, whether or not the test sample had precipitated was checked macroscopically. At the end of culturing, the number of cells was counted by using a Microcell counter (CDA-500; Sysmex, Hyogo, Japan) after trypsinization, and the cell growth rate at each dose was calculated. Finally, the prepared specimens were stained with Giemsa solution (Merck, Darmstadt, Germany) and 200 metaphase cells per dose were evaluated for structural aberrations and numerical aberrations, respectively.

##### 2.2.3.2 Evaluation of results

The *in vitro* chromosomal aberration test was considered positive when the frequency of cells with structural aberrations or numerically aberrant cells was 10% or more and the increase was dose-related. All other cases were considered negative. No statistical analyses were used.

## 3 Results

### 3.1 Analysis of SDs and STs in the test sample

**Fig. 1:**
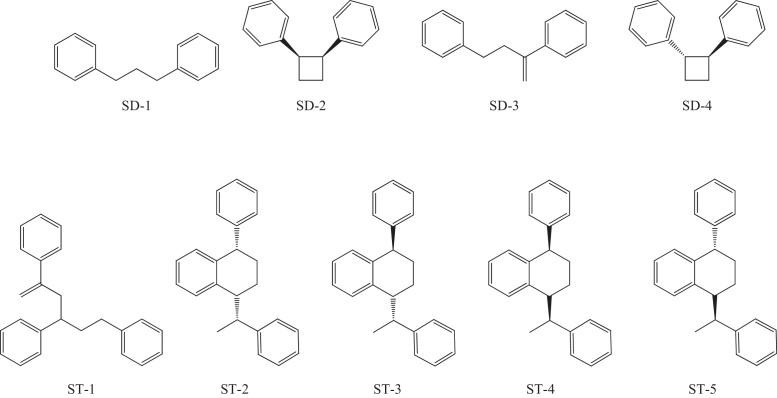
Chemical structures of styrene dimers and trimers detected in the test sample. Abbreviated names of each compound were designated in Table 3.

A total of four SDs and five STs were detected in the test sample (Table 3 and Fig. 1). The total SD and ST content of the test sample was approximately 88%; approximately 12% of the styrene oligomers in the test sample were of tetrameric or greater size (data not shown).

**Table 3:** Concentrations of styrene dimers and trimers detected in the test sample.

Compound name	Concentration (ppm)	Total concentration (ppm)
Styrene dimer		
1,3-Diphenylpropane (SD-1)	8	540
*cis*-1,2-Diphenylcyclobutane (SD-2)	77	
2,4-Diphenyl-1-butene (SD-3)	84	
*trans*-1,2-Diphenylcyclobutane (SD-4)	371	

Styrene trimer		
2,4,6-Triphenyl-1-hexene (ST-1)	1649	13,431
1e-Phenyl-4e-(1′-phenylethyl)tetralin (ST-2)	3078	
1a-Phenyl-4e-(1′-phenylethyl)tetralin (ST-3)	4821	
1a-Phenyl-4a-(1′-phenylethyl)tetralin (ST-4)	2114	
1e-Phenyl-4a-(1′-phenylethyl)tetralin (ST-5)	1769	

### 3.2 Ames test

**Fig. 2:**
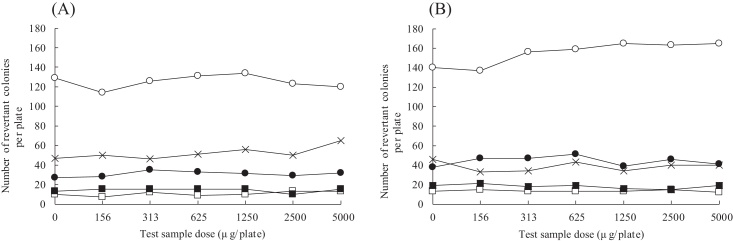
Dose–response curves in the Ames test in the absence (A) or presence (B) of S9 mix. ○, *Salmonella typhimurium* strain TA100; □, *S. typhimurium* strain TA1535; ●, *S. typhimurium* strain TA98; ■, *S. typhimurium* strain TA1537; ×, *Escherichia coli* strain WP2*uvrA*.

Prior to conducting the Ames test, a dose range-finding study was conducted using six doses: 156, 313, 625, 1250, 2500, and 5000 μg/plate. No increase in the number of revertant colonies and no bacterial growth inhibition were observed at any of the doses examined (data not shown). Therefore, the same six doses were used for the first Ames test. To confirm the reproducibility of the results, a second Ames test was conducted, but this time in consideration of the results of the first test, only five doses were used: 313, 625, 1250, 2500, and 5000 μg/plate. Because the dose–response curves obtained from the two Ames tests were comparable, only the dose–response curves from the first test are presented (Fig. 2).

The number of revertant colonies in each dose was similar to that in the negative control for all tester strains. No inhibition of bacterial growth and no precipitation of the test substance were observed for any of the test conditions in either the presence or absence of S9 mix. Therefore, because the number of revertant colonies in all treatment groups for all tester strains was less than twice that in each negative control in the presence or absence of S9 mix in both the first and second Ames test, the genotoxicity of the test solution was considered negative.

The numbers of revertant colonies in the negative control and positive control were within the historical range for our laboratory (data not shown).

### 3.3 *In vitro* chromosomal aberration test

Prior to the *in vitro* chromosomal aberration test, a dose range-finding study was conducted using nine concentrations: 19.5, 39.1, 78.1, 156, 313, 625, 1250, 2500, and 5000 μg/mL. No cytotoxicity was observed at any concentration under any condition, but precipitation of the test substance was observed at concentrations of 1250 μg/mL or greater. Therefore, only concentrations of 1250, 2500, and 5000 μg/mL were used in the *in vitro* chromosomal aberration test.

Relative cell growth rate was greater than 68% and no cytotoxicity was detected for all concentrations at all treatment conditions (Table 4). Precipitation of the test substance was detected at all three doses examined. The percentages of cells with structural aberrations or numerically aberrant cells were below 3% at all concentrations and for all treatment conditions; therefore, the *in vitro* chromosomal aberration test was considered negative for both structural and numerical aberrations.The frequencies of cells with structural aberrations in the negative and positive controls, and the frequencies of numerically aberrant cells in the negative control, were all within the historical range for our laboratory (data not shown).

**Table 4:** Results of the *in vitro* chromosomal aberration test.

Treatment condition	Test sample dose (μg/mL)	Precipitation from medium^a^	Relative cell growth rate (%)	Frequency of cells with aberrations (%)	
				Structural aberration	Numerical aberration
Absence of S9 mix (6-h treatment)	0	–	100	2.5	1.0
	1250	+	92.6	0.0	0.0
	2500	+	88.3	2.5	0.0
	5000	+	82.9	0.5	0.0

Presence of S9 mix (6-h treatment)	0	−	100	1.0	0.0
	1250	+	93.3	1.0	0.0
	2500	+	83.8	2.5	1.0
	5000	+	72.7	0.5	0.5

Continuous method (24-h treatment)	0	−	100	1.5	0.0
	1250	+	87.6	0.5	0.0
	2500	+	79.6	1.0	0.0
	5000	+	68.2	1.0	0.0

a Precipitation: − absence; + presence.

## 4 Discussion

There are few reports in the literature presenting evaluations of the genotoxicity of styrene oligomers. Grifoll et al. [16] reported a negative Ames test; however, their study examined only one tester strain (*S. typhimurium* strain TA98) under conditions of metabolic activation by the microsomal fraction of the livers of male Sprague-Dawley rats induced with Aroclor^®^ 1254. Therefore, the potential for extrapolating those results to determine the genotoxic effects of styrene oligomers on human health is limited. Thus, to contribute to the risk assessment of styrene oligomers migrated from polystyrene food packaging into food, in the present study we carried out the genotoxicity tests required by the FDA and EFSA for the safety evaluation of food packaging by using a concentrated solution of oligomers extracted from polystyrene intended for use in contact with food.

The migration of SDs and STs from polystyrene food packaging to food was investigated by Kawamura et al. [7], [8] and Nakada et al. [21]. The migration of SDs and STs to foods such as instant noodles under general use conditions has been investigated and compared with the concentrations of SDs and STs extracted with organic solvents [8]. The migration of SDs and STs to food can be as high as approximately 50 ppb [21], whereas the concentrations of SDs and STs extracted with 50% ethanol solution can be as high as 70 ppb (Table 5; [7], [8], [21]). The FDA recommends using 50% ethanol as a high-fat food simulant when examining the safety of polystyrene [3] and the EFSA recommends as milk products out of high-fat food simulant [15].

**Table 5:** Reported concentrations of styrene oligomers extracted from polystyrene food packaging with various simulants.

Extraction conditions	Concentration of styrene oligomer (ppb)		
Water, 95 °C, 30 min	ND^a^^,^^b^	ND^c^	0.04–4.0^d^
20% Ethanol, 60 °C, 30 min	5–10^b^	ND^c^	1.4–10.3^d^
50% Ethanol, 60 °C, 30 min	30–70^b^	ND–15.7^c^	–

a 60 °C, 30 min.

b Kawamura et al. [7], ND < 5 ppb.

c Kawamura et al. [8], ND < 1 ppb.

d Nakada et al. [21].

Reproductive toxicity testing in rats has shown that the extraction of SDs and STs from polystyrene with acetone allows rats to be exposed to doses of SDs and STs that are comparable to the daily intake in humans [13]. Therefore, in the present study, we also used acetone as the extraction solvent. However, this procedure might be given to leading an overestimation of the risk because it was expected that acetone had greater extraction capacity compared to the authentic simulant which represents the migration of styrene oligomers from polystyrene into food in general use. In this context, regardless of what the genotoxicity tests result in positive or negative, we can obtain the highest doses, at which the genotoxic responses become equivalent to the spontaneous levels. Then, to avoid overemphasis of the risk, we can compare these doses and the concentration of the styrene oligomers extracted by simulant and this comparison considering a margin of extraction amount would give us valuable insight to assess the risk of the styrene oligomers extracted from polystyrene.

Compared with 50% ethanol, acetone clearly had a greater capacity to extract SDs and STs. The concentrations of SDs and STs in the test solution were 540 ppm and 13,431 ppm, respectively (total, 13,971 ppm), whereas the maximum total concentration of SDs and STs that can be extracted from polystyrene with 50% ethanol solution is 70 ppb [7]. Therefore, the total concentration of SDs and STs extracted with acetone in the test solution was approximately 200,000 times higher than that extracted with 50% ethanol solution. This result shows that the capacities of solvents to extract styrene oligomers from polystyrene are influenced by the polarity of each solvent. Water, which possesses the highest polarity among solvents listed in Table 5, showed the lowest capacity to extract styrene oligomers. On the other hand, regarding the pattern of compounds extracted from the polystyrene, the ratio of SDs to STs became greater when the polarity of the solvent became higher [21].

In addition, the ratio of SDs to STs in the test solution used in the present study was not so different from that in the GPPS pellets themselves, showing that acetone extraction allowed cells to be exposed to test samples containing a sufficiently high concentration of SDs and STs in a similar ratio to that found in the original GPPS pellets.

Both the Ames test and the *in vitro* chromosomal aberration test, which are the tests required by the FDA and EFSA for the safety evaluation of food packaging, were negative even when high concentrations of oligomers were used compared to the case of fatty-food simulant, suggesting that the risk of genotoxicity of styrene oligomers migrated from polystyrene food packaging into food is very low.

Our results also provide useful data on the clastogenic and polyploidy-inducing potential of styrene oligomers. Because of the low solubility of styrene oligomers in aqueous condition, it was expected that the mixture of styrene oligomers would precipitate out of the culture medium during the *in vitro* chromosomal aberration test, which indeed occurred at doses of 1250 μg/mL or greater. As stated in OECD Guideline for the Testing of Chemicals, No. 473, regarding exposure conditions, doses with precipitation but no cytotoxicity should be used as the highest dose in the *in vitro* chromosomal aberration test. In the present study, all three concentrations assessed resulted in precipitation of the styrene oligomers out of the culture medium, confirming that the concentration of oligomers used in the present study contained high concentrations of styrene oligomers. In addition, there was no cytotoxicity at the three doses assessed. It is likely that the present results obtained by using an extracted solution of styrene oligomers are comparable to those that would be obtained by using a pure oligomer solution.

## 5 Conclusion

Our findings show the availability of acetone instead of 50% ethanol aqueous solution, which is recommended as a fatty-food simulant for polystyrene by FDA and EFSA, to extract styrene oligomers from polystyrene intended for use in contact with food to allow the evaluation of genotoxicity *in vitro*. Even if high concentrations were applied the Ames test and the chromosomal aberration test, styrene oligomers extracted from GPPS did not induce gene mutation nor chromosomal aberration, suggesting that the risk of the genotoxicity of styrene oligomers migrated from polystyrene food packaging into food is likely very low.

## Conflict of interest

The authors declare that there are no conflicts of interest.

## Transparency document

Transparency document
